# Risk of Clinically Relevant Pericardial Effusion After Pediatric Cardiac Surgery

**DOI:** 10.1007/s00246-018-2031-4

**Published:** 2018-12-11

**Authors:** Rik Adrichem, Saskia Le Cessie, Mark G. Hazekamp, Nicolette A. M. Van Dam, Nico A. Blom, Lukas A. J. Rammeloo, Luc H. P. M. Filippini, Irene M. Kuipers, Arend D. J. Ten Harkel, Arno A. W. Roest

**Affiliations:** 1 Division of Pediatric Cardiology, Department of Pediatrics, Leiden University Medical Center, PO Box 9600, 2300RC Leiden, The Netherlands; 2 Department of Medical Statistics and Bio-informatics, Leiden University Medical Center, Leiden, The Netherlands; 3 Department of Clinical Epidemiology, Leiden University Medical Center, Leiden, The Netherlands; 4 Department of Cardiothoracic Surgery, Leiden University Medical Center, Leiden, The Netherlands; 5 Division of Intensive Care, Department of Pediatrics, Leiden University Medical Center, Leiden, The Netherlands; 6 Division of Pediatric Cardiology, Department of Pediatrics, Free University Medical Center, Amsterdam, The Netherlands; 7 Division of Pediatric Cardiology, Department of Pediatrics, Juliana Children’s Hospital, The Hague, The Netherlands; 8 Department of Pediatric Cardiology, Academic Medical Center, Amsterdam, The Netherlands

**Keywords:** Congenital heart disease, Pediatric cardiac surgery, Postoperative pericardial effusion, Risk assessment

## Abstract

Pericardial effusion (PE) after pediatric cardiac surgery is common. Because of the lack of a uniform classification of the presence and severity of PE, we evaluated PE altering clinical management: clinically relevant PE. Risk factors for clinically relevant PE were studied. After cardiac surgery, children were followed until 1 month after surgery. Preoperative variables were studied in the complete cohort. Perioperative and postoperative variables were studied in a case–control manner. Patients with and without clinically relevant PE were matched on age, gender, and diagnosis severity in a 1:1 ratio. Multivariate analysis was conducted using important preoperative variables from the complete cohort combined with perioperative and postoperative variables from the case–control data. 1241 surgical episodes in 1031 patients were included. Clinically relevant PE developed in 136 episodes (11.0%). Multivariate correlation with the outcome was present for age, BSA (adjusted odds ratio: 1.6, 95% CI 0.9–2.8), right-sided heart defect (adjusted odds ratio: 1.3, 95% CI 0.9–1.9), history of previous operation (adjusted odds ratio: 0.5, 95% CI 0.3–0.7), cardiopulmonary bypass use (adjusted odds ratio: 2.1, 95% CI 0.9–4.5), duration of CPAP postoperatively, and an inotropic score (adjusted odds ratio: 1.01, 95% CI 0.998–1.03). In this large patient cohort, 11.0% of postoperative periods of pediatric cardiac surgery were complicated by PE requiring alteration of treatment. Secondly, we newly identified cardiopulmonary bypass use and right-sided heart defects as risk factors for clinically relevant PE and confirmed previously described risk factors: age, CPAP duration, BSA, and inotropic score and a previously described risk reductor: history of previous operation.

**Electronic supplementary material:**

The online version of this article (10.1007/s00246-018-2031-4) contains supplementary material, which is available to authorized users.

## Introduction

Congenital heart disease (CHD) occurs in about 12–14 per 1000 live births and is an important cause of pediatric mortality [1–3]. Due to improvements in detection of CHD and medical and surgical interventions, postoperative and long-term survival has increased [3, 4]. Yet, postoperative pericardial effusion (PE) remains a serious and frequent complication. PE occurs in approximately 20% of children undergoing cardiac surgery and is the cause of morbidity and hospitalization [5]. In 80% of cases, PE develops within 1 month after surgery [5]. In most cases, PE follows a relatively mild course that does not require intervention [6]. However, in severe cases, it can lead to cardiac tamponade, necessitating pericardiocentesis, and even death.

In children, an increased risk of developing any form of PE is associated with female gender, certain congenital defects, higher age at operation, and specific surgical procedures. Furthermore, higher pericardial drain production postoperatively and the use of anticoagulation were identified as a risk factor for PE after pediatric cardiac surgery [7, 8].

Research on PE after pediatric cardiac surgery is hampered by the lack of a standardized assessment of PE and its severity. Identification of high-risk patients for clinically relevant pericardial effusion (crPE), i.e., PE requiring additional echocardiograms, start or continuation of medication or pericardiocentesis, may refine patient management after pediatric cardiac surgery. Therefore, this study has two aims. The primary aim of the study is to evaluate the incidence of crPE after pediatric cardiac surgery in a tertiary center for CHD and to define preoperative, perioperative, and postoperative risk factors that are related to the occurrence of crPE.

## Materials and Methods

### Study Population

Patient files were reviewed of all patients that underwent a surgical procedure between January 2010 and December 2014 in the CAHAL center for CHD Amsterdam-Leiden, The Netherlands for the presence of crPE. CrPE was defined as PE that results in an alteration of the usual course of postoperative management, such as when additional echocardiographs were made, diuretics were initiated or continued, corticosteroid therapy was initiated or when pericardial drainage was required.

The CAHAL consists of three academic hospitals (Leiden University Medical Center, Academic Medical Center, and Free University Medical Center). All procedures took place in the department of cardiothoracic surgery of the Leiden University Medical Center. Patients were eligible for study inclusion when they were 18 years of age or younger at the time of surgery and when they underwent thoracic surgery of the heart and/or the great vessels.

### Data Collection

The EZIS patient information system (Chipsoft, Amsterdam, The Netherlands) and the HiX system (Chipsoft, Amsterdam, The Netherlands) were used to examine all files available in the LUMC. These files included all correspondence letters of the department of pediatric cardiology of the Leiden University Medical Center and of the intensive care department of the Leiden University Medical Center, as well as the letters of the collaborating hospitals. Intensive care information was thoroughly examined by means of the PDMS intensive care program (MetaVision iMDsoft, Düsselforf, Germany). Since 80% of patients develop PE within 1 month [5], 1 month of follow-up was collected for all patients. When patients were referred to the Academic Medical Center, the Free University Medical Center or the Juliana Pediatric Hospital, The Hague, The Netherlands within this month and letters of that period were not available in the EZIS system, patient files were examined at that location. When patients were referred to a different hospital, it was assumed they did not develop crPE, since patients with crPE would have been referred back to one of the hospitals of the CAHAL, because no specialized pediatric cardiologists are available in those centers.

Patients were examined in eligible surgical episodes. An eligible surgical episode was defined as the time interval between an eligible procedure, i.e., thoracic surgery of the heart and/or the great vessels, and 1 month of follow-up. Some patients underwent multiple procedures. When these procedures were performed within the period of 1 month, the follow-up was extended to 1 month after the last procedure. When procedures were performed more than 1 month apart, they were analyzed as separate episodes.

Routine echocardiographs were made at discharge from the intensive care unit (ICU), discharge from hospital and at least once during the second to fourth week after operation. Additional echocardiographies were made either during ICU stay or in between echocardiographies when a patient developed symptoms for which an echocardiograph was indicated. All echocardiography reports were examined to find out whether a patient developed PE during the episode and whether this PE was clinically relevant. For this, the echo-reports were searched for the mention of PE. When presence or absence of PE was not mentioned or if uncertainties existed within the report, a senior pediatric cardiologist (AAWR) reviewed the echocardiography. Patients were considered to have developed PE when there was echocardiographic evidence of effusion at least once during follow-up. As stated above, crPE was defined as PE that results in an alteration of the usual course of postoperative management, such as when additional echocardiographs were made, diuretics were initiated or continued, corticosteroid therapy was initiated or when pericardial drainage was required. The need for therapy initiation or adjustment was determined according to physicians’ discretion and distracted from the medical records. In these instances, the patients were considered to have developed crPE. Perioperative and postoperative patient data were collected until the moment that crPE was diagnosed.

### Potential Risk Factors for CrPE

Preoperative characteristics such as age at the time of procedure, gender, weight, length, and primary diagnosis were collected for all patients. Body surface area was calculated according to the formula of Dubois. Next, the primary diagnosis was classified as being simple, moderate, or severe in complexity according to the classification described by Hoffman and Kaplan [1]. This classification was used because it is a patient characteristic that conveys disease severity and because PE is known to occur more frequently in patients with specific congenital heart defects, such as tetralogy of Fallot, ventricular septal defect with additional defects, and isolated septal defects. Furthermore, for perioperative risk factors, we chose risk factors known from literature [9]: CPB use and duration and clamp time, clamp time. Therefore, we did not include classifications that focus more on difficulty of the performed operation, such as the STAT classification [10]. It was also noted whether the patient had a right-sided heart defect. These heart defects included atrial septal defect type 2, tricuspid valve disease, Ebstein tricuspid valve disease, tricuspid valve stenosis, tricuspid valve hypoplasia, sinus venosus atrial septal defect, double outlet right ventricle, and tetralogy of Fallot. An overview of the diagnoses included in this study with their severity and whether they were analyzed as being right-sided is given in Table S1.

To examine perioperative and postoperative characteristics, a case–control study was performed within the cohort. Therefore, all patients that developed crPE were selected and matched with patients that did not develop crPE in a ratio of one-to-one. Matching criteria were age, gender, and diagnosis severity. Matching was done in a two-step procedure. In the first step, a maximum age difference of 3 months was allowed. In the second step, cases that did not yet have a matched control were matched again with a maximum age difference of 12 months.

The perioperative characteristics that were investigated are shown in Table 4. If clamp time was not recorded, asystole duration as registered in the PDMS intensive care program was used instead. To justify this, clamp time was internally compared with asystole time in patients in whom clamp time and asystole time were both available. The maximum difference was 2 min, which was thought sufficiently small to justify the use of asystole time when clamp time was absent.

The postoperative characteristics that are investigated are also shown in Table 4. Inotropic use was assessed using a maximum inotropic score [11]. For respiratory ventilation, a distinction was made between bilevel positive airway pressure and/or assisted spontaneous breathing ventilation and continuous positive airway pressure ventilation. For corticosteroids use, perioperative use and the maximum dosage during ICU stay were recorded. Postoperative dexamethasone was only provided in case of stridor. However, due to few patients receiving corticosteroids, it was decided only to implement the use of peri- and postoperative corticosteroids in the statistical analysis. For diuretics, anticoagulation medication, antihypertensives, and non-steroidal anti-inflammatory drugs, it was only documented whether this type of medication was given and not the dose.

To prevent distortion of our results by outlying values, it was decided to categorize variables in which these outlying values could be expected. These variables were age, ICU duration, duration of bilevel positive airway pressure and/or assisted spontaneous breathing, and duration of continuous positive airway pressure ventilation. Clinically relevant cut-off values were chosen. Age was categorized into neonatal (0–1 month), 1–6 months, 6 months–1 year, and 1–18 years. ICU duration was categorized into ≤ 1 day, 1–5 days, 5–10 days, and > 10 days. Bilevel positive airway pressure and/or assisted spontaneous breathing ventilation was categorized into 0–1 h, 1–12 h, 12–24 h, and > 24 h. Continuous positive airway pressure ventilation was categorized into no continuous positive airway pressure usage, 0–1 h usage, 1–12 h, and > 12 h.

### Statistical Analysis

Preoperative patient characteristics were examined in the complete study cohort and perioperative and postoperative characteristics were assessed in the case–control sub-cohort. Continuous variables are shown as medians with interquartile ranges. Nominal variables are shown as frequencies and percentages. In the complete study cohort, patients with and without crPE were compared by means of independent sample student *T* tests for continuous variables and *χ*^2^ tests for dichotomous variables. Odds ratios were calculated by means of logistic regression analysis, with robust standard errors to account for clustered measurements within a child. The effect of perioperative and postoperative characteristics was assessed by comparing the patients with crPE to their matched controls, using paired *t* tests and McNemar tests. Conditional logistic regression was used to calculate odds ratios. Statistical significance was defined as *p* < 0.05.

The multivariate regression analysis was conducted in two steps. First the most predictive preoperative variables were selected. Therefore, the preoperative variables that were univariately associated with crPE (*p* value < 0.10) were entered in a logistic model. In the second step, the additional value of the peri- and postoperative variables was assessed using the case–control data. Unconditional logistic regression was used in which the regression coefficients for the preoperative variables were hold fixed at the values of the complete cohort analysis [12]. The peri- and postoperative variables that were univariately associated with crPE (*p* value < 0.10) were subsequently entered in the analysis. To account for missing data values, multiple imputation was used.

All analyses were conducted using SPSS statistics version 22.0 for Mac and STATA version 14 for Windows.

## Results

### Patient Characteristics

**Fig. 1:**
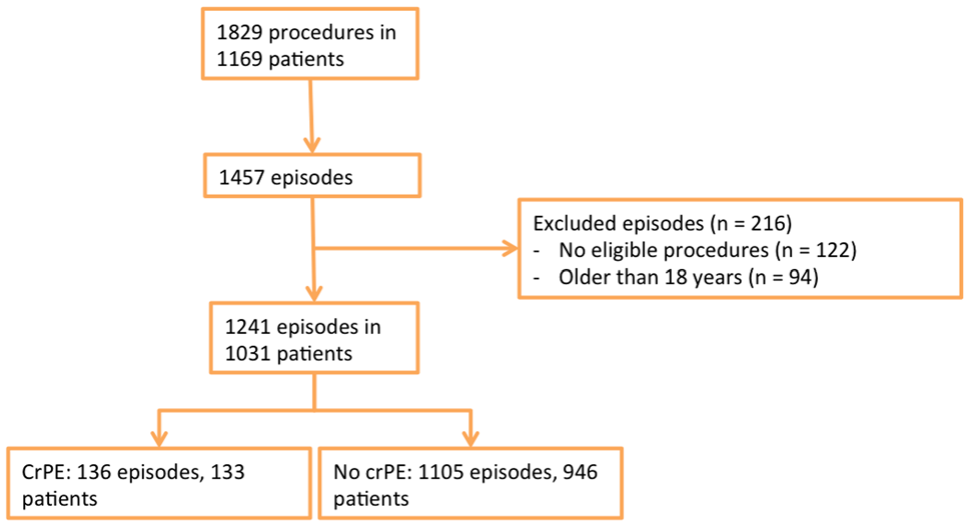
Flow chart of patient inclusion. *CrPE* clinically relevant pericardial effusion

During the study period, 1829 surgical procedures were performed on 1169 children, resulting in 1457 surgical episodes. Of these episodes, 1241 (1031 patients) were included because at least one eligible procedure was performed, i.e., thoracic surgery of the heart and/or great vessels with 1 month of follow-up (Fig. 1). Episodes were excluded because the patient was older than 18 years (*n* = 94) or because no eligible procedure was performed (*n* = 122). Of the 1031 patients, 863 patients (69.5%) had only one operation, and 168 had more than one operation. The characteristics of the included episodes are shown in Table 1.

**Table 1:** Patient characteristics at time of surgery in the included surgical episodes

Variable	Total episodes (*n* = 1241)^a^	
	*n* or median	% or IQR
Male	696	56.1%
Age at operation	8 months	2–49 months
0–1 month	213	17.2%
1–6 months	365	29.4%
6 months–1 year	124	10.0%
1–18 years	539	43.4%
Length	80 cm	62.0–112.0 cm
Weight	7.6 kg	4.3–15.8 kg
BSA	0.45 m^2^	0.30–0.73 m^2^
Previous operation	478	38.5%
Diagnosis severity		
Simple	197	15.9%
Moderate	419	33.8%
Severe	625	50.4%
Pericardial effusion	301	24.3%
Clinically relevant	136	11.0%

*BSA* body surface area, *IQR* interquartile range

^a^Missing values: length, *n* = 259; weight, *n* = 10; BSA, *n* = 261. Total patients missing ≥ 1 values, *n* = 261

Twenty-six patients died within the follow-up period. Of these, four developed PE, yet none of the deaths were caused by PE. One of these patients died of multi-organ failure with respiratory insufficiency, cardiac failure, and necrotizing enterocolitis; one died of treatment-resistant progressive bradycardia resulting in a failed resuscitation; one patient died of rhythm and output problems, complicated by brain hemorrhage, after which treatment was discontinued; and one patient died after discontinuation of treatment because of persistent necessity of respiratory support, progressive circulatory insufficiency, and absence of viable (surgical) treatment options. In two of the deceased patients, PE was evaluated as clinically relevant. One of these children was treated with diuretics, the other required pericardiocentesis.

### PE Incidence and Univariate Risk Factors

PE developed in 301 (24.3%) of the episodes, but in only 136 (11.0%), PE was considered clinically relevant. The 136 episodes with crPE occurred in 133 different patients. Twenty-seven patients had more than one surgical procedure during the course of this study, but only three patients developed crPE twice.

Table 2 shows the preoperative characteristics of the episodes with crPE compared with episodes in which no crPE developed and the univariate analysis of the preoperative variables. Of the preoperative factors, age, length, weight, right-sided heart defects, and body surface area were shown to increase crPE risk, while previous cardiac surgery was associated with a decreased risk of crPE (*p* < 0.05). Table 4 shows the results of the univariate analysis for the peri- and postoperative factors. Cardiopulmonary bypass use, bypass duration, clamp time, and CPAP duration were shown to be univariate risk factors for crPE.

**Table 2:** Comparison of preoperative characteristics between episodes with and without crPE

Variable	CrPE (*n* = 136)^a^		No crPE (1105)^b^		Univariate odds ratio (95% CI)		Multivariate odds ratio (95% CI)	
	*n* or median	% or IQR	*n* or median	% or IQR	Effect measure	95% CI	Effect measure	95% CI
Male	70	51.5%	627	56.7%	1.2	0.9–1.8		
Age at operation	15 months	4–85 months	7 months	1–46 months	1.004	1.002–1.007		
0–1 month	9	4.2%	204	95.8%	1		1.0	
1–6 months	35	9.6%	329	90.4%	2.4	1.1–5.1	2.2	1.0–4.8
6 months–1 year	20	16.1%	104	83.9%	4.4	1.9–9.9	4.1	1.7–9.9
1–18 years	72	13.3%	468	86.7%	3.5	1.7–7.1	3.1	1.3–7.2
Length	86 cm	67–124 cm	80 cm	62–109 cm	1.008	1.003–1.013		
Weight	9.45 kg	5.3–24.25 kg	7.5 kg	4.0–15.0 kg	1.015	1.005–1.025		
BSA	0.48 m^2^	0.32–0.94 m^2^	0.43 m^2^	0.29–0.71 m^2^	1.85	1.27–2.71	1.6	0.9–2.8
Previous operation	39	28.7%	439	39.7%	0.6	0.4–0.9	0.5	0.3–0.7
Diagnosis severity								
Simple	26	13.2%	171	86.8%	1			
Moderate	52	12.4%	368	87.6%	0.9	0.6–1.5		
Severe	58	9.3%	566	90.7%	0.7	0.4–1.1		
Right-sided heart defect	53	39.0%	322	29.1%	1.6	1.1–2.2	1.3	0.9–1.9

*BSA* body surface area, *crPE* clinically relevant pericardial effusion, *IQR* interquartile range

^a^Missing values: length, *n* = 15; weight, *n* = 2; BSA, *n* = 15. Total patients with ≥ 1 missing value(s), *n* = 15

^b^Missing values: length, *n* = 244; weight, *n* = 8; BSA, *n* = 246. Total patients with ≥ 1 missing value(s), *n* = 246

Of all episodes in which crPE developed, in 30 (22.1%) just an additional check-up was required. In the 106 other episodes, a treatment was started additive to the additional check-up: in 3 episodes (2.2%) non-steroidal anti-inflammatory drugs were started, in 44 (32.3%) diuretics were started, in 2 (1.5%) non-steroidal anti-inflammatory drugs as well as diuretics were started, in 25 (18.4%) corticosteroids were started, and in 31 (22.8%) pericardial drainage was required.

Most crPE occurred while the children were hospitalized, but in 26 episodes (19%) crPE developed after discharge. Details of these children are given in Table 3. Notably, 7 of these patients (26.9%) required pericardial drainage.

**Table 3:** Characteristics of specific subgroups of episodes in which crPE developed

Variable	CrPE development after discharge (*n* = 26)		Episodes requiring pericardial drainage (*n* = 32)	
	*n*	%	*n*	%
Male (%)	13	50.0	19	59.4
Age at operation				
< 3 years old	12	46.2	22	68.8
> 3 years old	14	53.8	10	31.2
Diagnosis severity				
Simple	5	19.2	2	6.3
Moderate	13	50.0	9	28.1
Severe	8	30.8	20	65.6
Initiated therapy				
Additional check-ups, *n* (%)	9	34.6	1	3.1
NSAIDs, *n* (%)	2	7.7	2	6.3
Diuretics, *n* (%)	9	34.6	13	40.6
Corticosteroids, *n* (%)	4	15.4	9	28.1
Pericardial drainage, *n* (%)	7	26.9	32	100

*crPE* clinically relevant pericardial effusion, *IQR* interquartile range, *NSAIDs* non-steroidal anti-inflammatory drugs

Of the 31 patients that went on to require pericardiocentesis, additional characteristics are presented in Table 3. Patients that did not receive corticosteroids for their crPE required drainage in 22.8% of the cases. Patients that did receive corticosteroids required drainage in 25.7% of the cases. This difference was not statistically significant (*p* = 0.71).

Twenty-one of the patients that required pericardial drainage were diagnosed with PE during routine echocardiography, either at the ward or at follow-up outpatient visits. Eleven patients developed symptoms because of their PE, which led to making additional echocardiographs, accelerating of the control visit, or admission to the hospital. Symptoms varied from increased irritability and seemingly infectious of origin to acute heart failure.

### Multivariate Analysis

Variables univariately associated (*p* < 0.10) with crPE were the preoperative factors age, length, weight, body surface area, history of previous operation, and right-sided heart defect; the perioperative factors cardiopulmonary bypass use, clamp time, and bypass duration, and the postoperative factors continuous positive airway pressure ventilation duration and the inotropic score. To prevent multicollinearity, length and weight were not used in the final model, because of the strong correlation with body surface area, and clamp time and bypass duration were not used because they were strongly related to cardiopulmonary bypass use. The first choice has been made, because it was felt that BSA did better convey the general state of health of a patient than did length, weight, or the combination of the two. The latter choice has been strengthened by the absence of a clear explanation of why bypass duration would increase the crPE incidence and because in our data, bypass use correlated better with the incidence of crPE than did clamp time or bypass duration.

The preoperative multivariate analysis is shown in Table 2. A multivariate analysis of the perioperative and postoperative factors, while taking into account the preoperative factors in the two-step manner described previously, yielded the results as shown in Table 4. In the multivariate analysis, age and CPAP duration significantly increased the risk for crPE and a history of previous surgery significantly reduced the risk. In addition, BSA, the use of cardiopulmonary bypass and intropics use were borderline significant (*p* < 0.10).

**Table 4:** Comparison of peri- and postoperative characteristics between episodes with and without crPE

	CrPE (*n* = 136)^a^		No crPE (135)^b^		Univariate odds ratio (95% CI)		Multivariate odds ratio (95% CI)	
	*n* or median	% or IQR	*n* or median	% or IQR	Effect measure	95% CI	Effect measure	95% CI
Perioperative variables								
CPB use	124	91.2%	103	76.3%	3.4	1.6–7.3	2.1	0.9–4.5
Bypass duration	94 min	58–150 min	74 min	0.0–111.0 min	1.006	1.002–1.011		
Clamp time	59.0 min	29.0–97.0 min	42.0 min	0.0–78.0 min	1.008	1.003–1.013		
Postoperative variables								
Inotropic score	12.0	4.8–21.0	8.00	1.0–15.0	1.02	0.998–1.033	1.01	0.998–1.03
Total corticosteroid use	65	47.8%	57	42.2%	1.4	0.79–2.6		
Anticoagulants use	27	19.9%	32	23.7%	0.79	0.44–1.4		
Diuretics use	111	81.6%	110	81.4%	1.00	0.51–2.0		
Antihypertensives use	10	7.4%	12	8.9%	0.82	0.34–2.0		
NSAIDs use	18	13.3%	16	11.9%	1.1	0.45–2.7		
Drain production								
0–100 mL	41	41.0%	59	59.0%	1			
100–200 mL	36	52.9%	32	47.1%	2.2	1.0–4.7		
200–500 mL	42	56.0%	33	44.0%	2.3	1.1–4.9		
> 500 mL	17	60.7%	11	39.3%	3.1	1.1–8.6		
PRISM III score	3.0	1.0–4.0	3.0	1.0–3.0	1.1	0.94–1.2		
PIM2 score	− 3.688	− 4.019 to − 3.295	− 3.710	− 4.318 to − 3.378	1.2	0.92–1.7		
BiPAP/ASB duration								
0–1 h	19	61.2%	12	38.8%	1			
1–12 h	50	47.6%	55	52.3%	0.57	0.26–1.2		
12–24 h	21	55.3%	17	44.7%	0.82	0.31–2.1		
> 24 h	46	47.4%	51	52.6%	0.53	0.21–1.3		
CPAP duration								
None	69	41.3%	98	58.7%	1		1.0	
0–1 h	24	47.1%	27	52.9%	1.8	0.84–3.9	1.5	0.7–2.9
1–12 h	22	84.6%	4	15.4%	16.3	3.4–77.4	10.8	3.3–35.2
> 12 h	21	77.8%	6	22.2%	8.9	2.4–33.9	4.4	1.5–12.8
ICU duration								
≤ 1 days	50	47.2%	54	52.8%	1			
1–5 days	48	49.5%	49	50.5%	1.1	0.63–2.0		
5–10 days	22	56.4%	17	43.6%	1.5	0.69–3.4		
> 10 days	16	51.6%	15	48.4%	1.3	0.54–2.9		
High urea	33	24.3%	31	23.0%	1.1	0.63–2.0		
High creatinine	11	8.1%	12	8.9%	1.1	0.35–3.3		

*ASB* assisted spontaneous breathing, *BiPAP* bilevel positive airway pressure, *CPAP* continuous positive airway pressure, *CPB* cardiopulmonary bypass, *crPE* clinically relevant pericardial effusion, *ICU* intensive care unit, *IQR* interquartile range, *NSAIDs* non-steroidal anti-inflammatory drugs

^a^Missing values: bypass duration, *n* = 2; clamp time, *n* = 5; inotropic score, *n* = 1; total corticosteroid use, *n* = 1; anticoagulants use, *n* = 1; NSAIDs use, *n* = 1; drain production, *n* = 2; PRISM III, *n* = 8; PIM 2, *n* = 8; BiPAP ASB duration, *n* = 2; highest urea, *n* = 4; highest creatinine, *n* = 4. Total patients with ≥ 1 missing value(s), *n* = 17

^**b**^Missing values: CPB use, *n* = 1; bypass duration, *n* = 23; clamp time, *n* = 2, PRISM III, *n* = 10; PIM 2, *n* = 10; IC duration, *n* = 5; highest urea, *n* = 8; highest creatinine, *n* = 8. Total patients with ≥ 1 missing value(s), *n* = 33

## Discussion

In this large single-center comprehensive evaluation of crPE after pediatric cardiac surgery, we show that crPE remains an important complication in the current era of pediatric cardiac surgery, complicating 11.0% of all surgeries. Univariate risk factors for crPE are older age at surgery, a higher body surface area, having a right-sided heart defect, cardiopulmonary bypass use, and a longer duration of continuous positive airway pressure ventilation postoperatively. A preventative factor is shown to be history of previous operation. However, in the multivariate analysis, only age and CPAP duration remained as significant risk factors and history of previous cardiac surgery as significant risk reductor.

### Risk Factors for CrPE

The findings regarding risk factors are mostly in accordance with previous literature, which also suggest an increased risk associated with older age in children [13–15] and preventative effect of previous cardiac surgery [9]. However, also some contradictions were found, since in this study no effect of gender or diagnosis severity could be identified and higher thoracic drain production, anticoagulation use, or longer intensive care stay did not seem to convey any additional risk [7, 8, 16]. In addition, this study identified cardiopulmonary bypass use and right-sided heart defects as new risk factors for crPE in children. It is hypothesized that right-sided heart defects and CPAP use both increase the risk for crPE by increasing the central venous pressure. A study by Elias et al. [17] recently investigated the risk for readmission for PE after pediatric cardiac surgery. In part, they found the same risk factors as were found in this study, such as higher age at surgery and atrial septal defect surgery, which in this study was grouped under the right-sided heart disease. In contrast, they also found that length of stay was a factor in determining the risk for PE and interventional procedures for PE. Our study adds a comprehensive analysis of patient files, instead of the analysis of an administrative database, with extra attention for the actual severity of PE because of our clinical outcome measure, i.e., crPE. Also, we had access to more detailed patient information, including perioperative and postoperative data in addition to the demographic information.

The pathophysiology of postoperative PE is still unclear, but there are various explanations. At least some part of the incidence is thought to be caused by the postpericardiotomy syndrome [6, 8, 13–15, 18–21], which is thought to be immune related. There are studies that support this claim by having identified antiheart antibodies and increases in pro-inflammatory cytokines [6, 13–15, 18–20, 22]. This hypothesis is also supported by studies investigating risk factors. In studies in children it is shown that very young age is associated with decreased crPE incidence [13] and in studies in adults it is shown that high age is associated with decreased incidence [9], which could be explained by the less-competent immune system in these patient groups [23]. Furthermore, one study in adults and one study in children show that female patients are more likely to develop PE compared to male patients [5, 8]. This also supports an influence of the immune system, since studies in children and adults show an increased incidence of auto-immune diseases in female patients [24, 25]. A third supportive finding for this hypothesis is the increased incidence of crPE related to cardiopulmonary bypass duration [9], since it is known that cardiopulmonary bypass use may trigger (systemic) immune reactions. A second pathophysiological mechanism could be related to blood in the pericardial space [7]. In this case, there might be an inflammatory response caused by the irritating effect of the blood [26]. This pathophysiologic mechanism may also be related to a different etiology of postoperative PE, namely the hemopericardium. In this study, there is insufficient data to distinguish hemopericardium and postoperative PE. However, we suspect the incidence to be low, a suspicion that is supported by the fact that anticoagulation use and high drain output were not found to be risk factors for crPE.

### Therapy for CrPE

PE requires intensive monitoring postoperatively by regular echocardiograms to start treatment at an adequate time and prevent complications such as cardiac tamponade and death. Prevention of PE has been studied by various studies [16, 21, 27–30]. Recently, the probability of using colchicine to treat and prevent postoperative PE has been intensely studied in adults, such as by the COPPS trials, the POPE-2 trial, and a study by Finkelstein et al. [28, 29, 31–33]. A meta-analysis by Imazio et al. [34] has also shown promising results in adults in preventing the postpericardiotomy syndrome as a cause of PE by means of colchicine therapy. However, at the moment, this effect has not yet been investigated in a pediatric patient population. In addition, this same meta-analysis looked at studies of Mott et al. [16] and Gill et al. [21], which investigated methylprednisolone and short acting acetylsalicylic acid as a preventative drug for postpericardiotomy syndrome in a pediatric population, but both studies reported no preventive effect of both medications. Therefore, there is not yet a proven preventative strategy for PE in a pediatric population.

Treatment options for PE are diverse and it is still unclear what is the best way to prevent or treat PE. Treatment options are fluid restriction, diuretic use, corticosteroid therapy, non-steroidal anti-inflammatory drugs, and, in case of cardiac tamponade, (surgical) pericardial drainage [35]. Compared to other pediatric studies investigating PE [7, 8, 13–15, 36], treatment of patients with crPE in this study was mainly conservative or with diuretics, with corticosteroids prescribed for more severe cases. Yet, corticosteroids did not seem to influence the progression of crPE, since patients with and without corticosteroids still required pericardial drainage just as often (22.8% vs. 25.7%, *p* = 0.71). This is in contrast with the results of the only randomized study in children that could be found regarding this subject and showed a significantly faster remission rate for PE in patients using corticosteroids [37]. However, that study is very small and only investigated 21 subjects. A large observational study by Pasquali et al. [38] investigating the overall usefulness of corticosteroids for PE in children also suggests no general benefit of corticosteroids. This study investigated the effect of corticosteroid therapy in 46,730 pediatric patients after CHD surgery in the United States of America and looked into mortality, increased hospital stay, and infection rates. Our findings also do not support the effect of corticosteroids on preventing pericardial drainage in patients with crPE and show that some children do not benefit from corticosteroid therapy.

### Strengths and Limitations

A strength of this study is the large patient population, which made it possible to accurately assess many possible risk factors. A second strength is that echocardiograms were routinely made in all postoperative patients. A third strength is that preoperative, as well as perioperative and postoperative factors were all explored. However, there are also some limitations to this study. Firstly, because of the retrospective study design, it was necessary to revert to patient charts, which might contain subjective descriptions and therefore it was not possible to objectively assess each patient. Secondly, there is no objective classification system to objectify the grade of PE for all patients. Therefore, we were obliged to rely on patient file descriptions of the PE and physicians interpretation of echocardiography results as to assess whether PE was clinically relevant. However, this resulted in a more clinical approach to the assessment of PE, which might be readily applied to patient care. Thirdly, only a random sample of matched controls was used in the case–control subpopulation to assess perioperative and postoperative factors. Fourthly, information on pericardial closure was mentioned only in the minority of surgical reports and therefore could not be investigated as a potential risk factor for crPE. Lastly, we did not investigate whether complexity of the surgery, as categorized by the STAT classification [10], conveys additional risk for PE. This classification has not been widely used in previous research, while literature in adult populations and pathophysiologic mechanisms show that CPB use and duration is associated with increased risk and is also related to the complexity of the surgery [9].

Additional research is required to fully understand all factors that play a role in the risk assessment for PE. It is expected that factors such as information on pericardial closure will be of additional value. A next step might be to form a prediction model identifying children at high risk for PE. This study created a first draft of such a model, which was associated with an area under the receiver-operator-characteristics curve of 0.71 and therefore as of yet insufficiently clinical applicable. The results of our efforts into creating a prediction model are shown in the supplementary materials.

In conclusion, this study confirms that several risk factors for PE that were known from adult literature also apply to children after pediatric cardiac surgery: cardiopulmonary bypass use and right-sided heart defect had not yet been shown to be risk factors in children. Furthermore, an important risk factor for crPE is shown to be older age and an important preventative factor is a history of previous surgery of the heart and/or great vessels.

## Electronic supplementary material

Below is the link to the electronic supplementary material.

Supplementary material 1 (DOCX 2475 KB)

Supplementary material 2 (DOCX 76 KB)

Supplementary material 3 (DOCX 58 KB)

## References

[1] Hoffman JI, Kaplan S (2002). The incidence of congenital heart disease. J Am Coll Cardiol.

[2] Dolk H, Loane M, Garne E (2011). Congenital heart defects in Europe: prevalence and perinatal mortality, 2000 to 2005. Circulation.

[3] Oster ME, Lee KA, Honein MA, Riehle-Colarusso T, Shin M, Correa A (2013). Temporal trends in survival among infants with critical congenital heart defects. Pediatrics.

[4] Vinocur JM, Menk JS, Connett J, Moller JH, Kochilas LK (2013). Surgical volume and center effects on early mortality after pediatric cardiac surgery: 25-year North American experience from a multi-institutional registry. Pediatr Cardiol.

[5] Imazio M, Brucato A, Rovere ME (2011). Contemporary features, risk factors, and prognosis of the post-pericardiotomy syndrome. Am J Cardiol.

[6] Imazio M (2012). The post-pericardiotomy syndrome. Curr Opin Pulm Med.

[7] Dalili M, Zamani H, Aarabi-Moghaddam M (2012). Pericardial effusion after pediatric cardiac surgeries: a single center observation. Res Cardiovasc Med.

[8] Cheung EWY, Ho SA, Tang KKY, Chau AKT, Chiu CSW, Cheung YF (2003). Pericardial effusion after open heart surgery for congenital heart disease. Heart.

[9] Ashikhmina EA, Schaff HV, Sinak LJ, Li Z, Dearani JA, Suri RM, Park SJ, Orszulak TA, Sundt TM (2010). Pericardial effusion after cardiac surgery: risk factors, patient profiles, and contemporary management. Ann Thorac Surg.

[10] O’Brien SM, Clarke DR, Jacobs JP (2009). An empirically based tool for analyzing mortality associated with congenital heart surgery. J Thorac Cardiovasc Surg.

[11] Klitsie LM, Hazekamp MG, Roest AAW, Van der Hulst AE, Gesink-van der Veer BJ, Kuipers IM, Blom NA, Ten Harkel ADJ (2013). Tissue Doppler imaging detects impaired biventricular performance shortly after congenital heart defect surgery. Pediatr Cardiol.

[12] Borgan Ø, Keogh R (2015). Nested case–control studies: should one break the matching?. Lifetime Data Anal.

[13] Engle MA, Mccabe JC, Ebert PA, Zabriskie J (1974). The postpericardiotomy syndrome and antiheart antibodies. Circulation.

[14] Engle MA, Zabriskie JB, Senterfit LB (1976). Heart-reactive antibody, viral illness, and the postpericardiotomy syndrome. Correlates of a triple-blind, prospective study. Trans Am Clin Climatol Assoc.

[15] Engle MA, Zabriskie JB, Senterfit LB, Gay WA, O’Loughlin JE, Ehlers KH (1980). Viral illness and the postpericardiotomy syndrome. A prospective study in children. Circulation.

[16] Mott AR, Fraser CD, Kusnoor AV, Giesecke NM, Reul GJ, Drescher KL, Watrin CH, Smith EO, Feltes TF (2001). The effect of short-term prophylactic methylprednisolone on the incidence and severity of postpericardiotomy syndrome in children undergoing cardiac surgery with cardiopulmonary bypass. J Am Coll Cardiol.

[17] Elias MD, Glatz AC, O’Connor MJ, Schachtner S, Ravishankar C, Mascio CE, Cohen MS (2016). Prevalence and risk factors for pericardial effusions requiring readmission after pediatric cardiac surgery. Pediatr Cardiol.

[18] Robinson J, Brigden W (1963). Immunological studies in the post-cardiotomy syndrome. Br Med J.

[19] Van Der Geld H, D’Ailly T (1964). Anti-heart antibodies in the postpericardiotomy and the postmyocardial-infarction syndromes. Lancet.

[20] McCabe JC, Ebert PA, Engle MA, Zabriskie JB (1973). Circulating heart-reactive antibodies in the postpericardiotomy syndrome. J Surg Res.

[21] Gill PJ, Forbes K, Coe JY (2009). The effect of short-term prophylactic acetylsalicylic acid on the incidence of postpericardiotomy syndrome after surgical closure of atrial septal defects. Pediatr Cardiol.

[22] Snefjellå N, Lappegård KT (2012). Development of post-pericardiotomy syndrome is preceded by an increase in pro-inflammatory and a decrease in anti-inflammatory serological markers. J Cardiothorac Surg.

[23] Simon AK, Hollander GA, McMichael A (2015). Evolution of the immune system in humans from infancy to old age. Proc R Soc B.

[24] Hon KE, Nelson EAS (2006). Gender disparity in paediatric hospital admissions. Ann Acad Med Singapore.

[25] Ngo ST, Steyn FJ, McCombe PA (2014). Gender differences in autoimmune disease. Front Neuroendocrinol.

[26] Pepi M, Muratori M, Barbier P, Doria E, Arena V, Berti M, Celeste F, Guazzi M, Tamborini G (1994). Pericardial effusion after cardiac surgery: incidence, site, size, and haemodynamic consequences. Heart.

[27] Imazio M, Belli R, Brucato A (2013). Rationale and design of the COlchicine for Prevention of the Post-pericardiotomy Syndrome and Post-operative Atrial Fibrillation (COPPS-2 trial): a randomized, placebo-controlled, multicenter study on the use of colchicine for the primary prevention of the postpericardiotomy syndrome, postoperative effusions, and postoperative atrial fibrillation. Am Heart J.

[28] Imazio M, Trinchero R, Brucato A (2010). COlchicine for the Prevention of the Post-pericardiotomy Syndrome (COPPS): a multicentre, randomized, double-blind, placebo-controlled trial. Eur Heart J.

[29] Meurin P, Lelay-Kubas S, Pierre B (2015). Colchicine for post-operative pericardial effusion. J Am Coll Cardiol.

[30] Sevuk U, Baysal E, Altindag R, Yaylak B, Adiyaman MS, Ay N, Beyazit U, Alp V (2016). Role of methylprednisolone in the prevention of postpericardiotomy syndrome after cardiac surgery. Eur Rev Med Pharmacol Sci.

[31] Imazio M, Brucato A, Ferrazzi P, Spodick DH, Adler Y (2013). Postpericardiotomy syndrome. J Cardiovasc Med.

[32] Mack DR, Cahoon WD, Lowe DK (2011). Colchicine for the primary prevention of the postpericardiotomy syndrome. Ann Pharmacother.

[33] Finkelstein Y, Shemesh J, Mahlab K (2002). Colchicine for the prevention of postpericardiotomy syndrome. Herz.

[34] Imazio M, Brucato A, Markel G, Cemin R, Trinchero R, Spodick DH, Adler Y (2011). Meta-analysis of randomized trials focusing on prevention of the postpericardiotomy syndrome. Am J Cardiol.

[35] Imazio M (2012). Contemporary management of pericardial diseases. Curr Opin Cardiol.

[36] Heching HJ, Bacha EA, Liberman L (2015). Post-pericardiotomy syndrome in pediatric patients following surgical closure of secundum atrial septal defects: incidence and risk factors. Pediatr Cardiol.

[37] Wilson NJ, Webber SA, Patterson MW, Sandor GG, Tipple M, LeBlanc J (1994). Double-blind placebo-controlled trial of corticosteroids in children with postpericardiotomy syndrome. Pediatr Cardiol.

[38] Pasquali SK, Hall M, Li JS, Peterson ED, Jaggers J, Lodge AJ, Marino BS, Goodman DM, Shah SS (2010). Corticosteroids and outcome in children undergoing congenital heart surgery: analysis of the pediatric health information systems database. Circulation.

