# Supplementary material for: Risk of Clinically Relevant Pericardial Effusion After Pediatric Cardiac Surgery
Source: Pediatr Cardiol. 2018 Dec 11;40(3):585–94. doi: 10.1007/s00246-018-2031-4 (PMC6420454; doi:10.1007/s00246-018-2031-4)

**Supplementary figure 1: ROC-curve of the model with only preoperative factors and of the final mode**


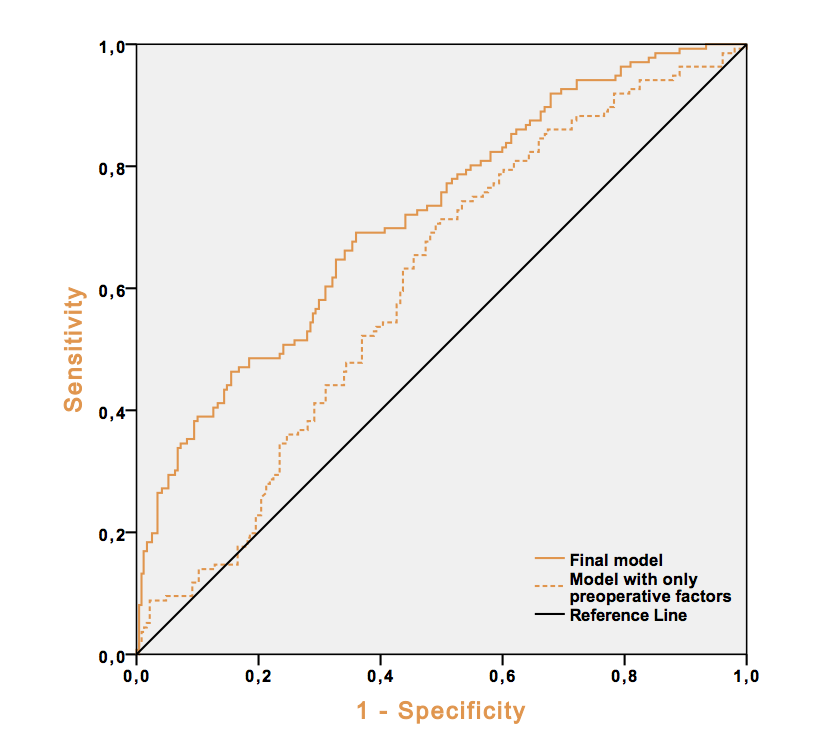


**Supplementary figure 2: Formula to calculate the propability of crPE in individual patients.**


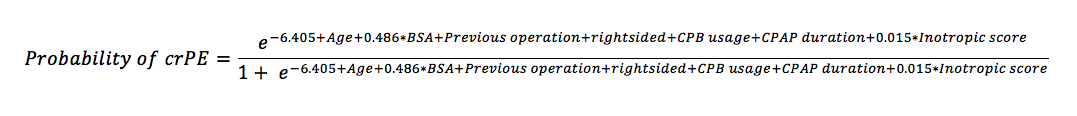

Supplement: Supplementary file 1 — Supplementary material 1 (DOCX 2475 KB) [file 246_2018_2031_MOESM1_ESM.docx]
