# Supplementary material for: Risk of Clinically Relevant Pericardial Effusion After Pediatric Cardiac Surgery
Source: Pediatr Cardiol. 2018 Dec 11;40(3):585–94. doi: 10.1007/s00246-018-2031-4 (PMC6420454; doi:10.1007/s00246-018-2031-4)
Supplement: Supplementary file 2 — Supplementary material 2 (DOCX 76 KB) [file 246_2018_2031_MOESM2_ESM.docx]

| **Supplementary table 1: Diagnosis and diagnosis severity** | | | | | | | | | | | |
| --- | --- | --- | --- | --- | --- | --- | --- | --- | --- | --- | --- |
| **Diagnosis severity** |  | **Total** | |  | **CrPE** | |  | **No crPE** | |  |  |
| **Simple** |  | n | % |  | n | % |  | n | % |  | **Rightsided** |
| **ASD type 2** |  | 56 | 4.5% |  | 13 | 9.7% |  | 43 | 3.9% |  | X |
| **Dilated cardiomyopathy** |  | 6 | 0.5% |  | - |  |  | 6 | 0.5% |  |  |
| **Mitral valve disease** |  | 13 | 1.0% |  | 3 | 2.2% |  | 10 | 0.9% |  |  |
| **PAPVR** |  | 8 | 0.6% |  | 2 | 1.5 |  | 6 | 0.5% |  |  |
| **PDA** |  | 22 | 1.8% |  | 1 | 0.7 |  | 21 | 1.9% |  |  |
| **Right coronary artery deviation** |  | 3 | 0.2% |  | - |  |  | 3 | 0.3% |  |  |
| **Arrhythmia** |  | 15 | 1.2% |  | 1 | 0.7 |  | 14 | 1.3% |  |  |
| **Arterial sling** |  | 2 | 0.2% |  | - |  |  | 2 | 0.2% |  |  |
| **Atrial thrombus** |  | 2 | 0.2% |  | - |  |  | 2 | 0.2% |  |  |
| **Myocardial tumor** |  | 3 | 0.2% |  | - |  |  | 3 | 0.3% |  |  |
| **Tricuspid valve disease** |  | 1 | 0.1% |  | - |  |  | 1 | 0.1% |  | X |
| **Superior vena cava deviation** |  | 1 | 0.1% |  | - |  |  | 1 | 0.1% |  |  |
| **Simple VSD** |  | 65 | 5.2% |  | 6 | 4.4% |  | 59 | 5.3% |  |  |
| **Moderate** |  |  |  |  |  |  |  |  |  |  |  |
| **ALCAPA** |  | 7 | 0.6% |  | 1 | 0.7% |  | 6 | 0.5% |  |  |
| **Aortic valve disease** |  | 66 | 5.3% |  | 16 | 11.8% |  | 50 | 4.5% |  |  |
| **ASD type 1** |  | 8 | 0.6% |  | - |  |  | 8 | 0.7% |  |  |
| **Aortic arch hypoplasia** |  | 7 | 0.6% |  | 1 | 0.7% |  | 6 | 0.5% |  |  |
| **Borderline LV hypoplasia** |  | 2 | 0.2% |  | - |  |  | 2 | 0.2% |  |  |
| **Coarctation of aorta** |  | 77 | 6.2% |  | 1 | 0.7% |  | 76 | 6.9% |  |  |
| **Ebstein tricuspid valve** |  | 1 | 0.1% |  | - |  |  | 1 | 0.1% |  | X |
| **HOCM** |  | 13 | 1.0% |  | 3 | 2.2% |  | 10 | 0.9% |  |  |
| **Left coronary obstruction** |  | 1 | 0.1% |  | - |  |  | 1 | 0.1% |  |  |
| **Pulmonary stenosis** |  | 15 | 1.2% |  | 2 | 1.5% |  | 13 | 1.2% |  | X |
| **Pulmonary valve hypoplasia** |  | 1 | 0.1% |  | - |  |  | 1 | 0.1% |  | X |
| **Sinus venosus ASD** |  | 23 | 1.9% |  | 2 | 1.5% |  | 21 | 1.9% |  | X |
| **Vascular ring** |  | 7 | 0.6% |  | 2 | 1.5% |  | 5 | 0.5% |  |  |
| **Valsalva aneurysm** |  | 11 | 0.9% |  | 1 | 0.7% |  | 10 | 0.9% |  |  |
| **VSD with concomitant defect** |  | 182 | 14.7% |  | 23 | 16.9% |  | 159 | 14.4% |  |  |
| **Severe** |  |  |  |  |  |  |  |  |  |  |  |
| **AVSD** |  | 93 | 7.5% |  | 19 | 14.0% |  | 74 | 6.7% |  |  |
| **AVSD with RV hypoplasia** |  | 1 | 0.1% |  | - |  |  | 1 | 0.1% |  |  |
| **Congenitally corrected TGA** |  | 12 | 1.0% |  | 4 | 2.9% |  | 8 | 0.7% |  |  |
| **Double inlet LV** |  | 15 | 1.2% |  | 1 | 0.7% |  | 14 | 1.3% |  |  |
| **DORV** |  | 77 | 6.2% |  | 9 | 6.6% |  | 68 | 6.2% |  | X |
| **DORV with LV hypoplasia** |  | 12 | 1.0% |  | - |  |  | 12 | 1.1% |  |  |
| **DORV with RV hypoplasia** |  | 1 | 0.1% |  | - |  |  | 1 | 0.1% |  |  |
| **Tetralogy of Fallot** |  | 106 | 8.5% |  | 8 | 5.9% |  | 98 | 8.9% |  | X |
| **HLHS** |  | 39 | 3.1% |  | - |  |  | 39 | 3.5% |  |  |
| **HRHS** |  | 4 | 0.3% |  | - |  |  | 4 | 0.4% |  |  |
| **Interrupted aortic arch** |  | 21 | 1.7% |  | 3 | 2.2% |  | 18 | 1.6% |  |  |
| **Left ventricle hypoplasia** |  | 6 | 0.5% |  | - |  |  | 6 | 0.5% |  |  |
| **Pulmonary atresia** |  | 75 | 6.0% |  | 3 | 2.2% |  | 72 | 6.5% |  |  |
| **Right ventricular hypoplasia** |  | 3 | 0.2% |  | - |  |  | 3 | 0.3% |  |  |
| **Tricuspid atresia** |  | 35 | 2.8% |  | 3 | 2.2% |  | 32 | 3.0% |  |  |
| **TAPVR** |  | 18 | 1.5% |  | - |  |  | 18 | 1.6% |  |  |
| **TGA** |  | 83 | 6.7% |  | 8 | 5.9 % |  | 75 | 6.8% |  |  |
| **Truncus arteriosus** |  | 22 | 1.8% |  | - |  |  | 22 | 2.0% |  |  |
| Abbreviations: ASD: atrium septum defect; PAPVR: partial abnormal pulmonary venous return; PDA: persistent ductus arteriosus; VSD: ventricular septum defect; ALCAPA: abberant left coronary artery to pulmonary artery; LV: left ventricle; HOCM: hypertrophied obstructive cardiomyopathy; AVSD: atrioventricular septum defect; RV: right ventricle; TGA: transposition of the great arteries; DORV: double outlet right ventricle; HLHS: hypoplastic left heart syndrome; HRHS: hypoplastic right heart syndrome; TAPVR: total abnormal pulmonary venous return. | | | | | | | | | | | |
