# Supplementary material for: Risk of Clinically Relevant Pericardial Effusion After Pediatric Cardiac Surgery
Source: Pediatr Cardiol. 2018 Dec 11;40(3):585–94. doi: 10.1007/s00246-018-2031-4 (PMC6420454; doi:10.1007/s00246-018-2031-4)
Supplement: Supplementary file 3 — Supplementary material 3 (DOCX 58 KB) [file 246_2018_2031_MOESM3_ESM.docx]

| **Supplementary table 2: Combined model preoperative and matched case-control results** | | | | | | | | | | | | | | |
| --- | --- | --- | --- | --- | --- | --- | --- | --- | --- | --- | --- | --- | --- | --- |
|  |  | | |  | | **Odds Ratio** | | | | | | |  |  |
| **Component of model** | **Regression coefficient** | | **S.E.** | | | | **Effect measure** | | **95% CI** | | | **p-value** | |  |
| Intercept | -6.405 | |  | | | |  | |  | | |  | |  |
| Preoperative factors, consisting of:  Age  0 – 1 month  1 month – 6 months  6 months – 1 year  1 – 18 years  BSA  History of previous operation  Right-sided heart defect | 0.0  0.798  1.417  1.124  0.486  -0.768  0.237 | | 0.389  0.445  0.430  0.284  0.213  0.202 | | | | 1.0  2.2  4.1  3.1  1.6  0.5  1.3 | | 1.0 – 4.8  1.7 – 9.9  1.3 – 7.2  0.9 – 2.8  0.3 – 0.7  0.9 – 1.9 | | | 0.04  <0.01  0.01  0.09  <0.01  0.24 | |  |
| CPB usage | 0.723 | | 0.396 | | | | 2.1 | | 0.9 – 4.5 | | | 0.07 | |  |
| CPAP duration  None  0-1 hours  1-12 hours  >12 hours | 0.0  0.374  2.382  1.479 | | 0.353  0.602  0.546 | | | | 1.0  1.5  10.8  4.4 | | 0.7 – 2.9  3.3 – 35.2  1.5 – 12.8 | | | 0.29  <0.01  0.01 | |  |
| Inotropic score | 0.015 | | 0.009 | | | | 1.01 | | 0.998 – 1.03 | | | 0.09 | |  |
| **Area under the curve** |  |  | | |  | | |  | | |  | | |  |
|  |  | | |  | |  | | | | **Proportion** | | | **95% CI** |  |
| Preoperative model only |  | | |  | |  | | | | 0.66 | | | 0.62 – 0.71 |  |
| Complete prediction model |  | | |  | |  | | | | 0.71 | | | 0.65 – 0.77 |  |
| Abbreviations: BSA: body surface area, CPB: cardiopulmonary bypass, CI: confidence interval, CPAP: continuous positive airway pressure, S.E.: standard error. | | | | | | | | | | | | | | |
